# Genetic support of a causal relationship between iron status and atrial fibrillation: a Mendelian randomization study

**DOI:** 10.1186/s12263-022-00708-9

**Published:** 2022-05-30

**Authors:** Tianyi Wang, Jun Cheng, Yanggan Wang

**Affiliations:** 1 Department of Cardiology, Zhongnan Hospital of Wuhan University, Wuhan University, Wuhan, China; 2 Department of Internal Medicine, Zhongnan Hospital of Wuhan University, Wuhan University, Wuhan, China; 3 Medical Research Institute of Wuhan University, Wuhan University, Wuhan, China

**Keywords:** Causal association, Iron status, Mendelian randomization, Atrial fibrillation

## Abstract

**Background:**

Atrial fibrillation is the most common arrhythmia disease. Animal and observational studies have found a link between iron status and atrial fibrillation. However, the causal relationship between iron status and AF remains unclear. The purpose of this investigation was to use Mendelian randomization (MR) analysis, which has been widely applied to estimate the causal effect, to reveal whether systemic iron status was causally related to atrial fibrillation.

**Methods:**

Single nucleotide polymorphisms (SNPs) strongly associated (*P* < 5 × 10^−8^) with four biomarkers of systemic iron status were obtained from a genome-wide association study involving 48,972 subjects conducted by the Genetics of Iron Status consortium. Summary-level data for the genetic associations with atrial fibrillation were acquired from the AFGen (Atrial Fibrillation Genetics) consortium study (including 65,446 atrial fibrillation cases and 522,744 controls). We used a two-sample MR analysis to obtain a causal estimate and further verified credibility through sensitivity analysis.

**Results:**

Genetically instrumented serum iron [OR 1.09; 95% confidence interval (CI) 1.02–1.16; *p* = 0.01], ferritin [OR 1.16; 95% CI 1.02–1.33; *p* = 0.02], and transferrin saturation [OR 1.05; 95% CI 1.01–1.11; *p* = 0.01] had positive effects on atrial fibrillation. Genetically instrumented transferrin levels [OR 0.90; 95% CI 0.86–0.97; *p* = 0.006] were inversely correlated with atrial fibrillation.

**Conclusion:**

In conclusion, our results strongly elucidated a causal link between genetically determined higher iron status and increased risk of atrial fibrillation. This provided new ideas for the clinical prevention and treatment of atrial fibrillation.

**Supplementary Information:**

The online version contains supplementary material available at 10.1186/s12263-022-00708-9.

## Introduction

Atrial fibrillation is the most common arrhythmia in clinical practice [1]. It is associated with a 5-fold risk of stroke and accounts for 15% of all stroke causes [2]. In addition, it is associated with a 2-fold risk of all-cause mortality [3], affecting the patient’s quality of life and increasing their economic burden. Iron is one of the essential elements for the human body [4]. It plays an important role in physiological processes, such as oxygen transport, immune function, electron transfer, energy production, and DNA synthesis [4]. Furthermore, iron catalyzes the formation of reactive oxygen species (ROS) and inflammatory factors, which may affect the occurrence of atrial fibrillation [5].

Some researchers have discovered that even if the left ventricle functions properly, atrial fibrillation can occur regardless of the iron status [6]. On the other hand, there is some evidence that iron overload significantly increases the chance of atrial fibrillation [7, 8]. However, inference of observational studies is limited by residual confounding, reverse causation, and detection bias [9]. Therefore, the link between iron status and the risk of atrial fibrillation still needs more attention.

Mendelian randomization uses genetics as an instrumental variable for exposure. While overcoming the limitations of traditional epidemiological research, it also strengthens causal inferences about the impact of specific exposure factors on the results [10]. Alleles are randomly distributed during gametogenesis, and genetic variations are randomly distributed in fertilized eggs. Genetic variation precedes the lifestyle and environmental factors selected by the individual, which minimizes the interference of confounding factors and overcomes the influence of reverse causality [11]. There are no MR-based studies to detect the relationship between iron status and the risk of atrial fibrillation. Therefore, we used public data from genome-wide association studies (GWASs) to investigate whether iron status may be causally related to the risk of AF.

## Methods

### Study design overview

**Fig. 1:**
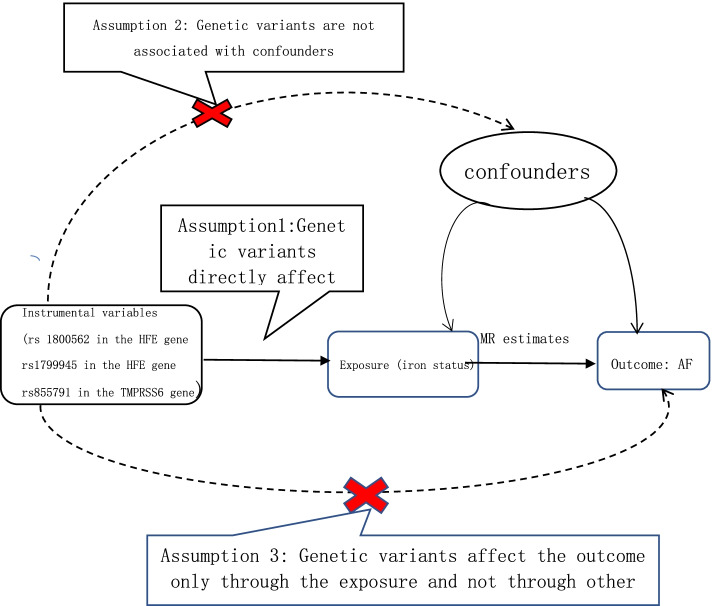
Flowchart of the instrument variables assumptions for MR design. SNP: Single nucleotide polymorphism

We conducted a two-sample MR study to investigate whether there is any causal effect of iron status on the risk of atrial fibrillation. Summary data come from GWAS consortia studies. The original studies were conducted with the informed consent of the participants, as well as ethical approval. We used SNPs as instrumental variables for the iron status. Key assumption methods include the following: (1) SNPs are related to the iron status (the exposure), (2) SNPs are independent of confounding factors, and (3) SNPs impact atrial fibrillation (the outcome) only through the iron status (the exposure) [12]. The overall study design is depicted graphically in Fig. 1.

To ensure the validity of the instrumental variables for MR analysis, the selection of instrumental variables needs to observe the following standards: single nucleotide polymorphisms (SNPs) are tightly related to the exposure of the genome-wide significance threshold (*p* < 5 × 10^−8^) [13]; linkage equilibrium is another necessary requirement for all SNPSs (pairwise *r*^2^ ≤ 0.01) [14]. Weak instrumental variables can also be associated with exposure factors, which can have an impact on MR research and lead to biases. The generation of weak instrumental variables is generally caused by an insufficient sample size. In general, some scholars have proposed evaluating the effect of weak instrumental variables through *F* statistical variables. From the perspective of traditional experience, it is generally better to have an *F* statistic greater than 10, and it is certainly better to have an *F* statistic greater than 100. When *F* statistic variables are less than 10, we usually consider that using genetic variation is a weak instrumental variable, which may produce certain bias. Hence, we need to be very careful when interpreting the results. Although some scholars believe that the *F* statistic may not be a very good instrument to evaluate the bias of weak instrumental variables, we still use the *F* statistic at this stage; after all, it is widely used and suggested to be a good method, while other new methods still need to be tested in practice. The formula for calculating the *F* statistic is as follows: *R*^*2*^*× (N – k − 1)/[(1 – R*^*2*^*) ×k]*. Here, *N* represents the GWAS research sample, *k* represents the number of independent variables (IV), and *R*^*2*^ is the IV explaining the exposed degree coefficient of the regression equation (decision). In a two-sample Mendelian randomization study, it is easy to obtain the specific values of *N* and *K*, but *R*^*2*^ is not easy to obtain, and we often need to refer to the original literature or the complete GWAS summary file to obtain it [15].

### Genetic associations with systemic iron status

To obtain summary-level data on the association between SNPs and iron status, the Genetics of Iron Status (GIS) consortium conducted a meta-analysis of the largest genome-wide association studies (GWASs), including 11 discovery cohorts and 8 replication cohorts, and a total of 48,972 European studies (46.9% for male participants) were included in the meta-analysis. This meta-analysis included a total of 19 cohorts and identified 12 SNPs related to biomarkers of systemic iron status at genome-wide significance (*p* < 5 × 10^−8^) (Table S1) and no linkage disequilibrium (LD) among them (all pairwise *r*^2^ ≤ 0.01). Five single nucleotide polymorphisms (SNPs) were associated with serum iron and transferrin saturation, six SNPs were associated with ferritin and eight SNPs were associated with transferrin [16]. Systemic iron status increased, indicating increased levels of serum iron, transferrin saturation and ferritin and decreased levels of transferrin [17]. Three of these 12 SNPs, rs1800562, and rs1799945 in HFE and rs855791 in TMPRSS6, showed a concordant change in four biomarkers of systemic iron status at genome-wide significance and accounted for most of the differences in each iron status biomarker [18]. Therefore, these three SNPs have sufficient effects to act as instrumental variables of iron status [19–21]. It seems unlikely that these biases are due to the effect of the weak instrumental variables because the value calculated by the *F* statistic variable is from 39 to 3340 [22]. The relationship between these SNPs and iron status biomarkers was obtained after the adjustment of covariables, including age and principal component scores, and other study-specific covariates. The cohorts included in the GIS consortium had a mean ± SD age ranging from 14.8 ± 2.2 to 68.2 ± 15.5 years. The proportion of female subjects was 53.1%.

Detailed information on SNPs and iron status is presented in Supplemental Tables S1 and S2 and is available at http://www.ncbi.nlm.nih.gov/pmc/articles/PMC421.

### Data sources: outcome

Summary-level data through the largest meta-analysis genome-wide association studies for AF (atrial flutter, paroxysmal AF, and persistent AF grouped together) were obtained from the AFGen (atrial fibrillation genetics) consortium study conducted by Roselli C et al. in 2018 [23]. To ensure that the effect assessments were consistent with the same alleles, they used the default settings for the harmonized data command in a two-sample MR package in R. This study contained information from more than 50 studies (84.2% European, 12.5% Japanese, 2% African American, and 1.3% Brazilian and Hispanic), including participants from UK Biobank, Biobank Japan, and included 65,446 atrial fibrillation cases and 522,744 controls. The data summary statistics can be obtained and downloaded from the two websites (http://afgen.org) and (http://www.kp4cd.org/datasets/v2f). The three instrumental variable SNPs related to iron status were available in the atrial fibrillation outcome GWAS.

### MR estimates

We mainly used the two-sample MR method to infer the causal association between iron status and AF. Therefore, we needed to use “Two sample MR” in the R package (version 0.4.23) to conduct MR analysis. Specifically, the inverse-variance-weighted (IVW) [24], MR–Egger regression [25], weighted median, simple mode, and weighted mode methods [26] were used to estimate the effect value between iron status and AF. Weighted median was the primary method to assess the association of genetically predicted iron status and AF risk. In addition, we present the results of different statistical methods in the same chart. The data we need for exposure and outcome were publicly available and accessible in the GWAS database. By using different methods, we finally obtained the three main results of beta, *SE* and *P* values through MR analysis. According to these results, we calculated 95% confidence intervals (CIs) and odds ratios (ORs). The formula required was as follows: OR = exp (beta); CI = exp (beta ± 1.96 × SE). During the analysis, it is precisely because of the existence of horizontal pleiotropic that results analysis may be difficult. We can obtain limited information through the GWAS catalog database (https://www.ebi.ac.uk/gwas). Through the retrieval of this website and literature reports, it was found that rs1799945 (HFE gene) has a certain relationship with high systolic and diastolic blood pressure [27]. The link between hypertension and atrial fibrillation is likely to lead to atrial fibrillation by dilating the left atrial diameter [28]. Then, we used the leave-one-out method to test the effect of rs1799945 (HFE gene) on the results.

### Sensitivity analyses

The purposes of our sensitivity analysis were to identify any potential possible pleiotropic and heterogeneous problems. Two of the assumptions in the MR analysis are that the instrumental variables could only influence AF by altering the selection of iron status markers, namely, the independence principle, and not by other confounders, namely, the exclusion-restrictions principle [29]. To exclude the heterogeneity of instrumental variables such as different experiments, platforms and populations, IVW and MR–Egger methods were used to test the heterogeneity. The value of each SNP and Cochran's Q statistics under each iron status were displayed through forest plots [30]. In addition, leave-one-out analysis was performed to identify SNPs with larger or nonproportional effects by removing one SNP at a time and recalculating estimates for the entire pool of instrumental variables. We used MR–Egger statistical sensitivity analysis to limit the pleiotropic effect of instrumental variables and make MR analysis more reliable. In the MR–Egger regression, the intercept, as an indicator of the mean multivariate bias, can be freely estimated [31]. Power calculations were based on a method designed for a binary outcome [32]. All of the above analyses were conducted using version 4.1.0 of R.

## Results

### Causal effects of iron status on the risk of AF

**Fig. 2:**
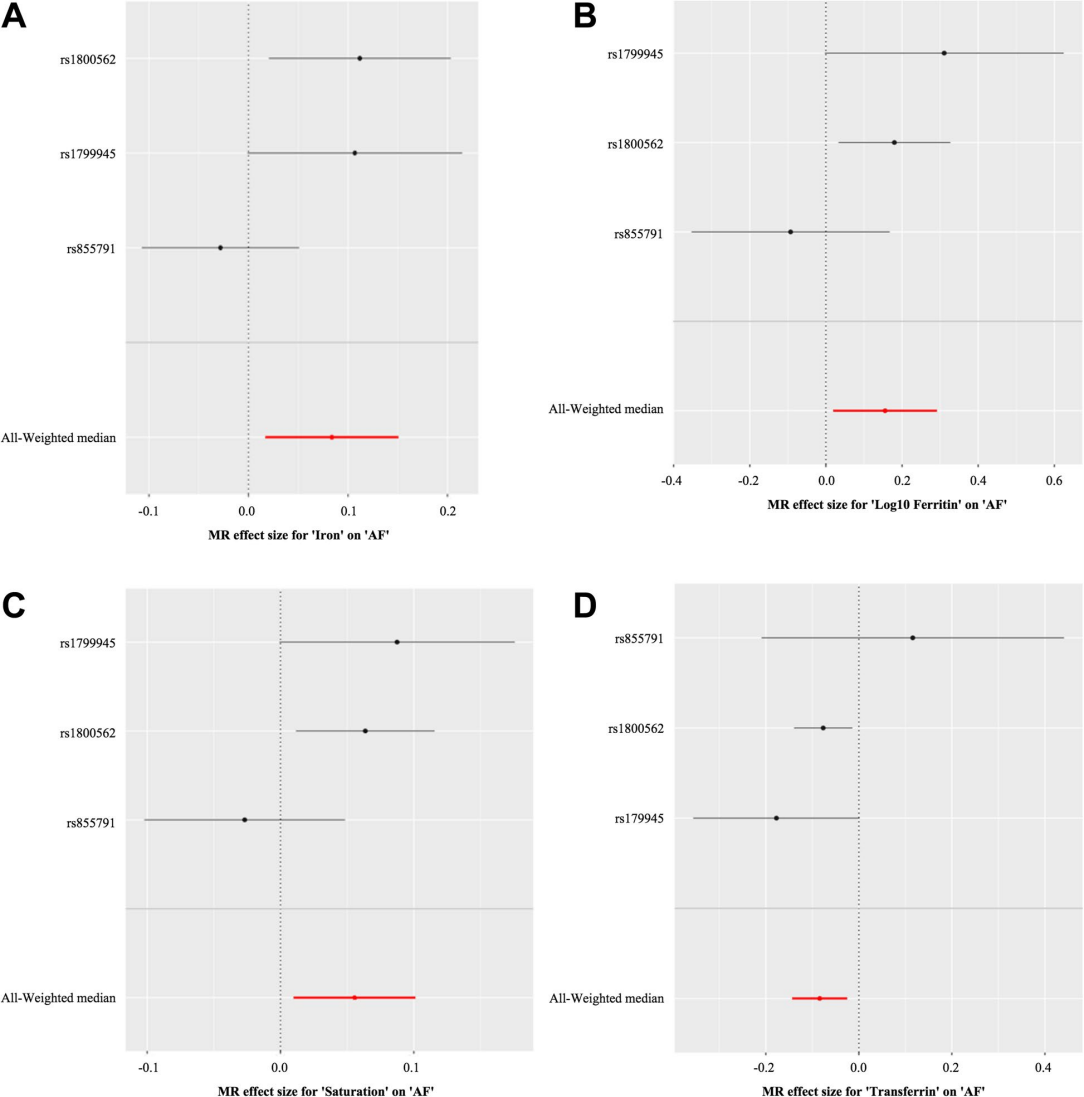
Forest plot of the specific SNP and pooled MR estimates for the causal effect of each iron status marker on AF risk (odds ratio [OR]). The size of the black horizontal lines reflects the precision of the MR estimates, and the size shows their 95% confidence intervals (95% CI) for each effect allele. The pooled MR estimate is depicted by the red horizontal lines, with the size indicating the 95% CI. **a** Iron-AF, **b** Log10 Ferritin-AF, **c** Saturation-AF. **d** Transferrin-AF

The MR analysis results are shown as odds ratios (ORs) for AF per standard deviation (SD) increase in each biomarker of iron status and risk of AF. We found that serum iron [OR 1.09; 95% confidence interval (CI) 1.02–1.16; *p* = 0.01], ferritin [OR 1.16; 95% CI 1.02–1.33; *p* = 0.02], and transferrin saturation [OR 1.05; 95% CI 1.01–1.11; *p* = 0.01] had significant positive effects on AF. However, higher transferrin levels [OR 0.90; 95% CI 0.86–0.97; *p* = 0.006] were associated with a lower probability of AF, which is indicative of decreased iron status. The relationships between each biomarker of iron status and the risk of AF are shown graphically in Fig. 2.

### Sensitivity analyses did not display indication of unknown pleiotropy

**Fig. 3:**
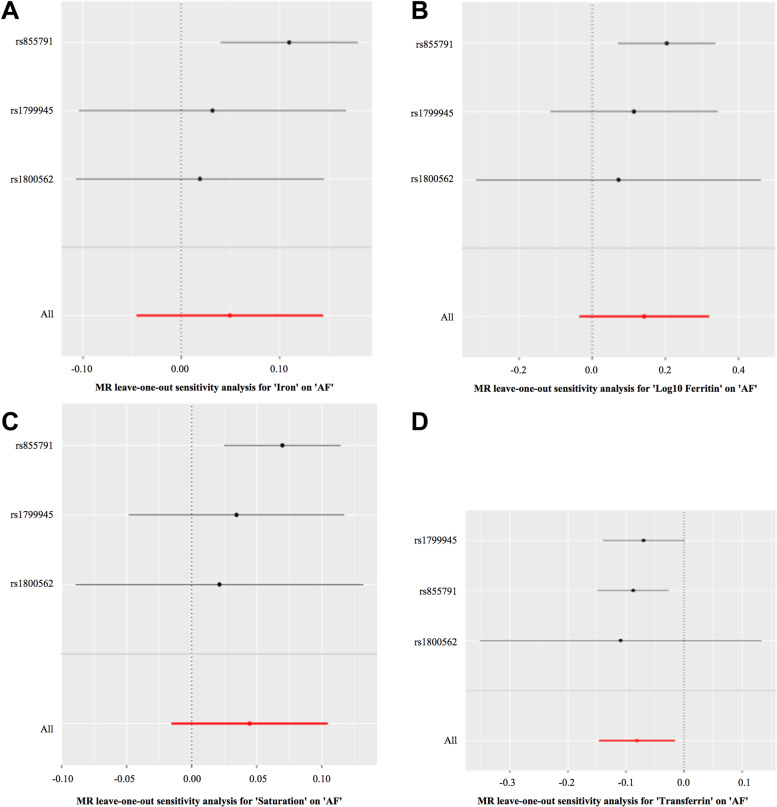
Plots of “leave-one-out” analyses for MR analyses of the causal effect of iron status on AF in replicative practice. **A** Iron-AF. **B** Log10 Ferritin-AF. **C** Saturation-AF. **D** Transferrin-AF

The presupposition of Mendelian randomization study for causality inference is that there is no level of pleiotropy biases. We used the PhenoScanner database to examine the biological pleiotropy of these instruments to evaluate the possible biases [33]. As expected, three SNPs affect red blood cell traits by changing the iron status [34]. The MR–Egger intercepts for the four biomarkers of iron status for directional horizonal pleiotropy did not differ significantly from null (*p* = 0.51, 0.65, 0.55, and 0.79 for serum iron, ferritin, transferrin saturation, and transferrin, respectively). The MR–Egger for the four biomarkers for the heterogeneity test did not differ significantly from null (*p* = 0.07, 0.07, 0.09, 0.13, and for serum iron, ferritin, transferrin saturation, and transferrin, respectively). Some articles pointed out that the iron status-raising alleles at rs1800562 (HFE gene) and rs1799945 (HFE gene) were associated with lower low-density lipoprotein levels and higher systolic and diastolic blood pressures [35]. After searching a large number of existing studies, there was no clear relationship between hypolipidemia and atrial fibrillation. Although one of the alleles was associated with high blood pressure, which can induce AF, we conducted a leave-one-out sensitivity analysis and found that there were no changes in the MR estimate. Even if the direction of estimates varied somewhat (*P* > 0.05), it did not change the pattern of results in Fig. 3.

**Fig. 4:**
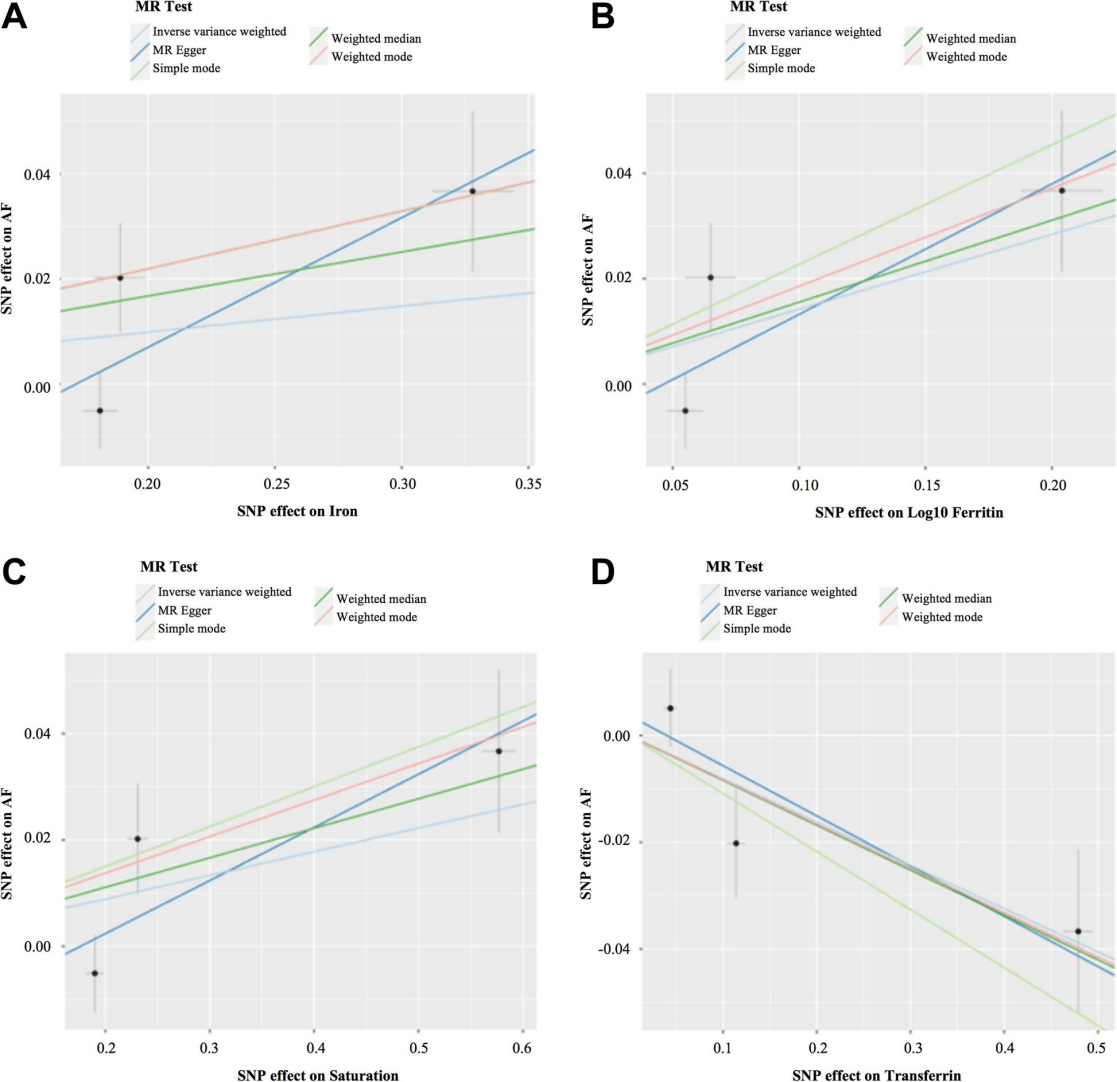
Scatter plots for MR analyses of the causal effect of iron status on AF in initial practice. **A** Iron-AF. **B** Log10 Ferritin-AF. **C** Saturation-AF. **D** Transferrin-AF. The slope of each line corresponds to the estimated MR effect per method

MR analysis was carried out using five methods. MR–Egger, weighted median, simple mode, and weighted mode produced directionally consistent effects as the IVW estimates. We compared the five MR analyses in different iron statuses and charted them in Fig. 4.

## Discussion

Previously, there have been observational studies of iron status in relation to arrhythmias and atrial fibrillation [36]. Excess or lack of nutrients can lead to many chronic diseases [37]. Due to the differences in race, ethnicity, and sample size, as well as some unconsidered confounding factors or unknown risk factors, the observation results easily exhibit biases. Consequently, we used two-sample MR, which can minimize the interference of confounders, to estimate the associations between several genetically determined markers of systemic iron status (serum iron, ferritin, transferrin saturation, and transferrin) and the risk of AF. The basic assumption of two-sample MR is that the instruments (SNPs) should be associated with the outcome (AF) only via the exposure (systemic iron status as reflected by the four iron biomarkers). Furthermore, we use summary level data generally mainly from the largest meta-GWAS of European descent to reduce the likelihood of biases [38]. The final results showed that iron overload is associated with an increased risk of AF based on MR analysis. Moreover, the conclusions obtained by different MR analysis methods are consistent. These results provide a reasonable way to use iron status as a promising clinical target for AF prevention and treatment [39].

The reliability of the results may not be stable due to the pleiotropic effects of the MR analysis method [40]. We used SNPs in the PubMed database to search for the possibility of secondary phenotypes. The association of the 3 iron status instruments is a well-established relationship between iron status and anemia [16]. However, if any effect of RBC traits on AF risk was downstream of iron status, instead of independent of it, this would not bias the MR analysis [41]. Our online search identified two SNPS—rs1800562 and rs1799945—in the HFE gene. The former is associated with lower low-density lipoprotein levels, and the latter is associated with higher systolic and diastolic blood pressures. The influence of blood lipids on atrial fibrillation has not been clearly reported. However, blood pressure has been reported to increase the risk of AF [28]. By causing left ventricular hypertrophy, atrial dilatation is more likely to lead to atrial fibrillation [42–44].

Nevertheless, removing the SNP produced no substantive effect in MR analysis results, suggesting that the MR estimates in the present study were not likely to be expected to be biased by blood pressure. Due to the influence of different MR analysis methods, the slight differences in the confidence interval width and estimates may be explained by accident, possibly, the results of difference measurement errors rather than by distinct real differences. In addition, using the MR–Egger method to conduct the pleiotropic test did not detect biases and associations. Both exposure and outcome in the public genome-wide association study database were mainly from the European population, thus minimizing the biases of population stratification. Meanwhile, the leave-one-out MR estimate was parallel to the primary MR estimates. Generally, it shows that there were unlikely to be serious biases in our research methods and conclusions.

Iron deficiency and systemic iron overload can cause metabolic disorders. Approximately one-third of patients with heart failure and one-half of patients with pulmonary hypertension have iron deficiency. Moreover, iron deficiency has an adverse effect on patients with coronary artery disease, heart failure, pulmonary hypertension, and patients who may undergo heart surgery [45]. An increase in systemic iron status means that serum iron, transferrin saturation and ferritin levels rise, while transferrin levels decrease [46]. Previous MR studies have shown that increased iron status reduces the risk of coronary artery-related diseases [47] and Parkinson’s syndrome [48] and increases the risk of type 2 diabetes [49] and cardiogenic thrombosis [21]. To the best of our knowledge, no causal relationship has been conducted previously between instruments’ iron status and atrial fibrillation using MR methods. Hence, we suggest a causal relationship between systemic iron status and AF for the first time.

The most important risk factors for the occurrence of atrial fibrillation events are aging, hypertension, diabetes, atrial dilation and left atrial enlargement, stroke, cardiomyopathies, heart failure and genetics [50]. In addition, natriuretic peptide levels and volume overload also increase the probability of atrial dilation and atrial fibrillation [51]. Our MR study found that one of the alleles related to iron status was rs1799945 (HFE gene), which is related to high systolic blood pressure and high diastolic blood pressure [27, 52]. As mentioned above, hypertension is one of the risk factors for atrial fibrillation, which is a confounding factor leading to the effect of iron status on AF. Through leave-one-out pleiotropic analysis, the results did not change significantly. When iron overload is determined by the cardiac magnetic resonance (CMR) value, the risk of atrial fibrillation is significantly increased [53]. Whether there are potential mechanisms by which iron overload directly affects the occurrence of AF remains to be determined. We obtained clues from some observations and basic experimental results.

First, in addition to the abovementioned volume overload being the main reason for atrial dilation, another major factor is oxidative stress [54]. Iron overload is an important cause of oxidative stress [55]. Oxidative stress can induce changes in intracellular calcium ions, leading to delayed depolarization and resulting in atrial focal ectopic action potential [56]. In addition, abnormal calcium ion handling and calcium overload could induce cardiac remodeling, resulting in atrial fibrillation [57]. When the iron overload exceeds the iron storage capacity, erratic iron enters the circulation [5] and can also penetrate into the myocardial cells [55]. Iron overloaded cardiomyocytes have abnormal action potentials compared with normal cells. Animal experiments have found that iron toxicity can lead to changes in the electrical conduction of the heart and arrhythmia [58]. Iron toxicity damages cardiomyocyte conduction by increasing oxidative stress. Oxidative stress disrupts the balance of sodium, potassium, and calcium channels, resulting in abnormal cell ion flow, insufficient atrial contraction, and atrial fibrillation [55]. Another mechanism is that oxidative stress activates nuclear factor-κB (NF-κB). NF-κB can downregulate calcium channels and lead to atrial fibrillation [59]. In an experiment on older rats, iron overload caused oxidative stress and activated NF-κB in brain tissue [5]. In addition, excess free ions can participate in the Fenton reaction and produce a large amount of OH^-^, which is considered to be the strong activation of ROS [60, 61]. In 2012, Dixon et al. discovered a new method of programmed cell death—ferroptosis—the essence of which is the Fenton reaction [62]. Thus, it can be fully inferred that iron overload may cause atrial fibrillation through ferroptosis. It has been proven in animal experiments that ferroptosis occurs in atrial fibrillation, and inhibiting ferroptosis can effectively control the occurrence of atrial fibrillation [63]. Finally, some studies have found that in thalassemia patients, iron overload is caused by ineffective hematopoiesis and repeated blood transfusions [64, 65]. In these patients, inflammatory factors are elevated. By using iron chelating agents, the levels of inflammatory factors demonstrate a downward trend [66]. Therefore, iron overload can cause changes in the heart through inflammatory factors, making the heart more prone to atrial fibrillation, and it is one of the regulators of inflammation [57]. In summary, iron status can lead to atrial fibrillation through potential mechanisms: oxidative stress, inflammatory response, and ferroptosis. Our findings provide the first evidence that four genetically determined systemic iron status biomarkers (serum iron, ferritin, ferritin saturation, ferritin) are significantly associated with atrial fibrillation risk. This was consistent with some previously recognized associations between iron and AF based on observational studies.

Herein, our research should be interpreted in consideration of some limitations. First, because our analyses were conducted in publicly available GWAS databases, it was difficult to perform stratified analyses, such as age and sex, using the exposure and outcome databases. Furthermore, even though we used different methods to try to minimize the pleiotropy and data obtained from the GWAS database with the largest sample size in the world, biases caused by unknown biological effects of the SNP on iron status may be inevitable. Moreover, the datasets used in our research were mainly derived from European populations, which aimed to reduce the bias owing to race. Hence, it is unclear whether the results are appropriate for other races. Finally, further research is needed to investigate the causal effects of iron status on AF risk in subjects with severe iron overload or deficiency. Despite these limitations, the results of this research show consistent and biologically plausible effects.

Ultimately, some Mendelian randomization studies used IV-W as the main method of analysis, while we used weighted median for the following reasons to make the results more reliable and reasonable. As a valid IV, there are three necessary assumptions: (IV1) the variant is predictive of the exposure; (IV2) the variant is independent of any confounding factors of the exposure-outcome association; and (IV3) the variant is conditionally independent of the outcome given the exposure and the confounding factors [67]. Of these, only IV1 was verified (*p* < 5 × 10^−8^), while the rest depended on all possible confounding factors of exposure and outcome, both measured and unmeasured. As Mendelian randomization analysis contains multiple variants, statistical capacity is improved [68]. However, one of the challenges is that not all included genetic variants are valid IVs [69]. If all genetic variants satisfy the IV assumptions, IVW can adequately reflect the real causality. However, when an invalid IV occurs, the IVW deviates from the true causality, resulting in a bias outcome [70]. Voight et al. suggested that there is not even a moderate causal effect of HDL-c on CAD risk [71]. In this study, we found that one of the IVs may be an invalid or weak instrument (it is associated with the confounding factor of hypertension), and therefore, using the IVW method is not appropriate. The weighted median methods generally have more power with a positive causal effect, especially when the proportion of invalid IVs increases, and have fewer mean standard errors than the IVW method [70]. Therefore, for this study, weighted median methods are statistically closer to the real causality.

## Conclusion

Through the MR method, we tested the previously assumed hypothesis that there is a causal relationship between the overload in the iron status of the systematic and the occurrence of AF. We relied on iron status data measured in 48,972 individuals in the general population database and the AFGen (Atrial Fibrillation Genetics) consortium study, including participants from UK Biobank, Biobank Japan, and included 65,446 atrial fibrillation cases and 522,744 controls in the general population to conduct a two-sample MR experiment. We used the three SNPs as instruments to increase statistical power by combining their MR estimates and investigate possible pleiotropic effects. Our Mendelian randomization study first showed that systemic iron status increases AF risk. This has important clinical significance for the treatment of borderline anemia and the continued iron therapy of anemia patients after the iron status is corrected, especially for patients with anemia or continuous supplementation for patients at high risk of atrial fibrillation, and careful consideration may be needed.

## Supplementary Information

**Additional file 1: Table S1.** Iron status on genome-wide significance level biomarker-related SNPs and included in all three main SNPs and secondary analysis of the main analysis. **Table S2.** Additional information was amplified for three major SNPs.

## Data Availability

All relevant data supporting the conclusions are included in this published article and its supplementary information files.

## Abbreviations

AF: Atrial fibrillation
MR: Mendelian randomization
SNP: Single nucleotide polymorphisms
GWAS: Genome-wide association studies
NF-κB: Nuclear factor-κB
HDL: High-density lipoprotein
ROS: Reactive oxygen species
AFGen: Atrial Fibrillation Genetics
CMR: Cardiac magnetic resonance
ORs: Odds ratios
SD: Standard deviation
CI: Confidence interval
IVW: Inverse-variance-weighted
LD: Linkage disequilibrium
GIS: Genetics of Iron Status
IV: Independent variable
